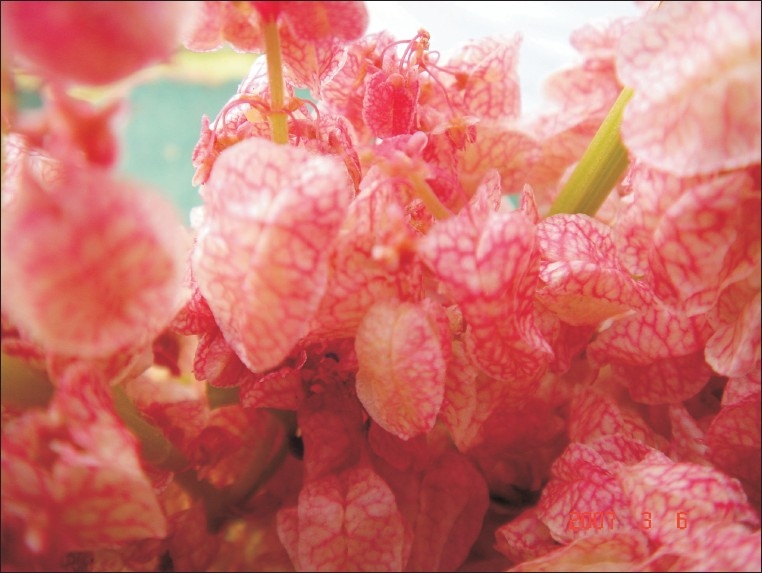# Tranquility of nature

**Published:** 2008

**Authors:** Ammar Ibne Anwar

**Affiliations:** Guest Faculty, Faculty of Unani Medicine, Aligarh Muslim University., K-78, Safina Apartment, Medical Road, A.M.U., Aligarh-202 002, U.P., India. Member of Indian Red Cross Society, Member of National Safety Management Society, U.S.A. Patron of vanco. Health society, B.C., Canada, E-mail: ammaramu@yahoo.com

**Figure:**